# Post-traumatic stress disorder in parents of patients with schizophrenia following familial violence

**DOI:** 10.1371/journal.pone.0198164

**Published:** 2018-06-01

**Authors:** Masako Kageyama, Phyllis Solomon

**Affiliations:** 1 Department of Health Promotion Science, Osaka University Graduate School of Medicine, Suita, Osaka, Japan; 2 School of Social Policy & Practice, University of Pennsylvania, Philadelphia, Pennsylvania, United States of America; Technion Israel Institute of Technology, ISRAEL

## Abstract

The present study conducted in Japan aimed to clarify the relationship between violence directed towards parents by patients with schizophrenia and parents’ risk of post-traumatic stress disorder (PTSD). Questionnaire data from 353 parents were analyzed. In total, 84 of the 353 parents (23.8%) reported the Impact of Event Scale-Revised (IES-R) score ≥ 25 (high-IES-R), indicative of a high risk of developing PTSD. The rate of high-IES-R scores was significantly higher among parents who had experienced an act of violence that was likely to result in severe injury by their adult child with schizophrenia (OR = 2.03; 95% CI 1.09–3.80; using “never experienced” as a reference) and in parents of patients who were hospitalized at the time of the survey (OR = 2.47; 95% CI 1.01–6.06; using “regularly visited a psychiatrist” as a reference). Therefore, parents experiencing violence by their adult child with schizophrenia are at a risk of developing PTSD. Parents of patients with schizophrenia, who are at a high risk of PTSD, are not usually provided the required support in Japan. To prevent violence and provide support for family members who may develop PTSD, it is necessary to establish crisis intervention programs, especially given the current emphasis on deinstitutionalization policy in Japan.

## Introduction

In Japan, the 12-month prevalence of post-traumatic stress disorder (PTSD) is reported to be between 0.4% [1] and 0.7% [2] in the general population. According to a community survey in Japan, approximately 60% of the respondents reported exposure to at least one traumatic event in their lifetime. For individual events, a higher conditional risk of PTSD was observed in those who had been beaten (5.7%), raped (5.6%), or had a child with a serious illness (4.4%) [2]. The experience of violence and having a child with a serious illness appears to have a significant impact on the risk of PTSD in the Japanese population.

Parents with an adult child with a serious mental illness are at risk for experiencing serious violence perpetrated by their child. In Japan, the Medical Treatment and Supervision Act [3] for persons with mental illness who have committed serious criminal offences such as homicide or serious injury was enacted in 2005. Under this law, special involuntary treatment services in designated hospitals or in the community are provided for such persons. Most patients under involuntary treatment orders have been those diagnosed with schizophrenia (77.0%) [4]. Half of all violent acts committed by those with severe psychiatric disorders was found to be directed at family members [5].

The impact of Event Scale-Revised (IES-R) is a screening questionnaire for PTSD risk [6]. In Japan, 39% of family members of inpatients and outpatients with schizophrenia are reported to have IES-R scores ≥ 25 (high-IES-R) [7], and 58% of parents or siblings of patients who had undergone long-term hospitalization had high-IES-R scores [8]. One of the reasons for a high PTSD risk rate is thought to be violence in the home [7,8]. However, the evidence regarding the relationship between familial violence and a high risk of PTSD in family members of patients with schizophrenia has not been demonstrated.

A high risk of PTSD in parents of patients with schizophrenia is an issue of great concern because parents may be likely to fear repeated violence, and consequently, may resist patients being discharged from the hospital. Japan has the highest psychiatric bed ratio among developed countries [9]. Although the Japanese government has implemented a significant number of policies promoting deinstitutionalization, these policies have not been particularly successful. One of the reported challenges of deinstitutionalization is that the anxiety of family members frequently results in their resistance to the discharge of their patient relative [10]. We suspect that in the past the patient’s parents and other family members may have experienced violence by the patient which may have resulted in their anxiety regarding the patient returning home. As a national survey found that 65% of patients with serious mental illness live with parents [11], parents have an increased likelihood of experiencing violence. Consequently, we hypothesized that there is a significant relationship between violence experienced by parents and their risk of developing PTSD.

The current study focused on parents of patients with schizophrenia, as the majority (almost 60%) of patients in inpatient settings in Japan are diagnosed with schizophrenia [12]. Thus, this population is impacted most by the recent deinstitutionalization policy. The present study, therefore, aimed to clarify the relationship between violence directed towards parents by patients with schizophrenia and parents’ risk of developing PTSD. This study has the potential for important implications for the discharge planning process and the prevention of familial violence.

## Materials and methods

### Study sample and data collection

The present analysis was part of a larger study, “Japanese Family Violence and Mental Illness” [13]. The larger study aimed to examine the prevalence of familial violence and related factors among caregivers and siblings in 866 households belonging to 27 affiliate family groups under a prefectural-level family group association in Japan.

Questionnaires were distributed to 768 of the 866 households in the group association. The distribution of questionnaires was determined by the group leaders. Questionnaires were not distributed to 118 households due to health conditions or family issues. Of the 482 returned caregiver questionnaires (from 350 households), 463 were valid (346 households). The present analysis focused on caregiver questionnaires completed by caregivers of patients with schizophrenia. The sample size for this analysis was 353 after the exclusion of questionnaires regarding patients diagnosed with illnesses other than schizophrenia (n = 43), respondents other than parents (n = 22), and those with missing IES-R data (n = 59) (with overlap, n = 110 excluded).

### Measures

#### Risk of PTSD as the dependent variable

Risk of PTSD was evaluated using the IES-R, a 20-item screening questionnaire focused on experiences within the past seven days [6]. Scores can range between 0 and 80. A higher score represents a higher risk of PTSD. The reliability and validity of the Japanese version of the IES-R has a sensitivity of 75–89% and a specificity of 71–93% for screening for risk of PTSD and has an estimated best cut-off point of 24/25 [14]. The Cronbach’s alpha in the current study was 0.97.

#### The experience of violence as an independent variable

No standard measure of violence for families with a relative with a severe psychiatric disorder exists; therefore, items regarding the violence that parents experienced were constructed and divided into three sections. First, 14 items were created from preliminary qualitative data from parent interviews regarding their experience of violence. The lifetime frequency of certain behaviors was selected from the following options: “never,” “1–4 times,” “5–99 times,” or “100 times or more.” Second, the operational definitions of violent experiences were specified in reference to those found in the literature. In the current study, psychological violence was defined as the use of verbal or non-verbal communication to cause another person mental or emotional harm, and physical violence was defined as the use of physical force with the potential for causing death, disability, injury, or harm [15]. The category of psychological violence included five items (Table 1): shouting (1), blaming “my illness is your fault” (2), swearing and insulting (3), saying “I will kill you” (4), and punching or kicking gestures (5). Acts of physical violence were divided into two categories: “acts of violence” and “other aggressive acts” based on the categorization used in the MacArthur Violence Risk Assessment Study (MVRAS) [16]. “Acts of violence” were operationally defined as acts that resulted in physical injury or were likely to result in severe injury and were committed by using a weapon or by choking. This category included five items (Table 1): visit to a physician resulting from injury (10), knife injury (11), threatening with a knife (12), beating with an object (13), and choking (14). “Other aggressive acts” were operationally defined as acts that did not result in injury or were not likely to result in severe injury and were committed without the use of a weapon or by choking; these included four items (Table 1): destroyed property (6), pushing (7), punching and kicking (8), and throwing an object (9). Third, responses were categorized as the existence of violence if any item from the psychological violence list occurred 5 times or more in a lifetime, and if any item from the acts of violence or other aggressive acts lists occurred once or more.

**Table 1:** Relationship of parents’ experiences of violence by their adult child with schizophrenia and their risk of PTSD (N = 353).

No. item or category	All	Low-IES-R (≤ 24)	High-IES-R (> 25)	p
	n = 353	n = 269	n = 84	
Type of violence	n (%)	n (%)	n (%)	
1 shouting (> 5 times)	171 (48.6)	116 (43.1)	55 (66.3)	.0002
2 blaming ‘my illness is your fault’ (> 5 times)	86 (24.4)	54 (20.1)	32 (38.6)	.0006
3 swearing and insulting (> 5 times)	108 (30.7)	69 (25.7)	39 (47.0)	.0002
4 saying ‘I will kill you’ (> 5 times)	24 (6.8)	12 (4.5)	12 (14.5)	.0016
5 gesturing of punching or kicking (> 5 times)	81 (23.0)	48 (17.8)	33 (39.8)	< .0001
psychological violence (1–5; ≥ 5 times in any item)	198 (56.1)	135 (50.2)	63 (75.0)	< .0001
6 destroyed property	236 (67.1)	172 (63.9)	64 (77.1)	.0257
7 pushing	132 (37.5)	88 (32.7)	44 (53.0)	.0008
8 punching and kicking	140 (39.8)	94 (34.9)	46 (55.4)	.0009
9 throwing an object	113 (32.1)	78 (29.0)	35 (42.2)	.0246
other aggressive acts (6–9; ≥ 1 time)	261 (74.2)	189 (70.3)	72 (86.8)	.0027
10 visited physician for injury	38 (10.8)	21 (7.8)	17 (20.5)	.0011
11 injured with knife	9 (2.6)	3 (1.1)	6 (7.2)	.002*
12 threatening with knife	53 (15.1)	30 (11.2)	23 (27.7)	.0002
13 beating with an object	49 (13.9)	26 (9.7)	23 (27.7)	< .0001
14 choking	21 (6.0)	13 (4.8)	8 (9.6)	.1155*
acts of violence (10–14; ≥ 1 time)	100 (28.4)	60 (22.3)	40 (48.2)	.0027

*Fisher’s exact test.

#### Control variables

Relationship to the patient (father or mother), age, caregiver status (i.e., primary or not primary), and cohabitation with the patient were measured as factors for the parents. Gender, age, years since disease onset, current psychiatric care, medication use as instructed, number of hospitalizations, and use of rehabilitation services were measured as factors for the patients. All items are in S1 and S2 Questionnaires.

### Data analysis

Initially, we confirmed the normality of the variables employing descriptive statistics by assessing the distribution of each variable. Next, the frequency distribution of the IES-R scores and violence were computed. The violence items and the background characteristics in the high- and low-IES-R groups were compared using t-tests, chi-square tests, or Fisher’s exact tests. We used t-tests for continuous variables, chi-square tests for categorical variables in which each cell had an expected frequency of five or more, or Fisher’s exact tests for categorical variables in which one or more cells had an expected frequency of less than five. Finally, to examine the relationship between IES-R and the type of violence experienced, a multiple logistic regression was performed, with the high- and low-IES-R groups as the dependent variable, the types of violence as the independent variables, and other variables related to the dependent variable at the *p* < .20 level of significance as control variables. We tested for multicollinearity using the variance inflation factor (VIF) and confirmed that VIF was < 2.0 among the selected variables. All analyses were conducted using SAS Version 9.4 (SAS, Cary, NC).

### Ethical considerations

The Research Ethics Committee of the Faculty of Medicine of the University of Tokyo approved this study, including its consent procedure (February 24, 2014; No. 10415). All participants were informed of the study’s aim and that participation was voluntary. Informed consent was implied through questionnaire completion and return. Although we used identification numbers for the particular family group to which we distributed the questionnaires, we ensured that confidentiality of the collected data and anonymity of respondents were maintained by not using an identification number or any code in such a way that they could be linked to a specific household or individual’s name. The contact information for agencies that could provide assistance to participants who required help pertaining to familial violence was given.

## Results

### Demographic data of parental respondents and their relative with schizophrenia

Demographic data of parental respondents is shown in Table 2. Two-thirds of the respondents were mothers (68.0%). The respondents had an average age of 69 ± 7.3 years. Almost three-quarters of the respondents (70.2%) were primary caregivers, and most lived with the patient (84.3%). Two-thirds of the patient relatives were male (63.3%) and had an average age of 38.8 ± 7.7 years. They had lived with schizophrenia for an average of 18.5 years. Most of them (87.2%) visited a psychiatrist regularly and less than 10% (8.5%) of them were hospitalized at the time of questionnaire completion. Less than 20% of the patients had never been hospitalized. Most of them took medication as prescribed (94.0%). Almost half (45.7%) of the patients spent their time at home without the benefit of rehabilitation services.

**Table 2:** Demographic data of parents and their patient relative by high and low-IES-R groups.

			All	Low-IES-R (≤ 24)	High-IES-R (> 25)	
			n = 353	n = 269	n = 84	
		n	n (%) Mean±SD	n (%) Mean±SD	n (%) Mean±SD	*P*
Parents’ factors						
Relationship	Father	353	113 (32.0%)	90 (33.5%)	23 (27.4%)	.297
	Mother		240 (68.0%)	179 (66.5%)	61 (72.6%)	
Age (years)	Average	343	69.0±7.3	68.9±6.8	69.3±8.9	.661
	Under 60		33 (9.6%)	21 (8.0%)	12 (14.8%)	.052
	60–69		146 (42.6%)	117 (44.6%)	29 (35.8%)	
	70–79		135 (39.4%)	106 (40.5%)	29 (35.8%)	
	80 or older		29 (8.4%)	18 (6.9%)	11 (13.6%)	
Primary caregiver	Yes	346	243 (70.2%)	179 (68.1%)	64 (77.1%)	.116
	No		103 (29.8%)	84 (31.9%)	19 (22.9%)	
Cohabitation with patient	Yes	350	295 (84.3%)	228 (85.4%)	67 (80.7%)	.307
	No		55(15.7%)	39 (14.6%)	16 (19.3%)	
Patients’ factors						
Gender	Male	346	219 (63.3%)	164 (62.1%)	55 (67.1%)	.416
	Female		127 (36.7%)	100 (37.9%)	27 (32.9%)	
Age (years)	Average	347	38.8±7.7	38.8±7.5	39.1±8.5	.734
	Under 30		39 (11.2%)	29 (11.0%)	10 (12.1%)	.957
	30–39		147 (42.4%)	114 (43.2%)	33 (39.8%)	
	40–49		137 (39.5%)	103 (39.0%)	34 (41.06%)	
	50 or older		24 (6.9%)	18 (6.8%)	6 (7.2%)	
Years since onset		346	18.5±8.2	18.2±8.0	19.7±8.7	.139
Psychiatrist visit	Regularly	351	306 (87.2%)	241 (90.3%)	65 (77.4%)	.002
	Hospitalized		30 (8.5%)	15 (5.6%)	15 (17.8%)	
	Not regularly		15 (4.3%)	11 (4.1%)	4 (7.8%)	
Medication as prescribed	Yes	351	332 (94.6%)	256 (95.9%)	76 (90.5%)	.092*
	No		19 (5.4%)	11 (4.1%)	8 (9.5%)	
Number of hospitalizations	0		68 (19.4%)	56 (20.9%)	12 (14.4%)	.082
	1–2		169 (48.2%)	133 (49.6%)	36 (43.4%)	
	3 or more		114 (32.5%)	79 (29.5%)	35 (42.2%)	
Rehabilitation	Yes	348	159 (45.7%)	135 (50.9%)	24 (28.9%)	.0004
	No		189 (54.3%)	130 (49.1%)	59 (71.1%)	

1): Conversion of 100 JPY to US$1.

*P* values were calculated for the differences between high- and low-IES-R groups using the t-test, chi-square test, or Fisher’s exact test (*).

SD: Standard deviation.

### IES-R and familial violence

A total 84 of the 353 parents (23.8%) had IES-R scores of 25 or higher (high-IES-R group), while 269 (76.2%) had scores of 24 or lower (low-IES-R group).

In order of descending prevalence, the rate of experiencing each type of psychological violence 5 times or more in a lifetime was as follows: shouting (48.6%), swearing and insulting (30.7%), blaming “my illness is your fault” (24.4%), punching or kicking gestures (23.0%), and saying “I will kill you” (6.8%). On all items, parents with high IES-R scores had significantly more experiences of violence than parents with low IES-R scores. The percentage of parents who selected 5 or more in any item of psychological violence was 56.1%.

In order of descending prevalence, the rate of experiencing each type of “other aggressive acts” was as follows: destroyed property (67.1%), punching and kicking (39.8%), pushing (37.5%), and throwing an object (32.1%). On all items, parents with high IES-R scores had significantly more experiences of violence than parents with low IES-R scores. The percentage of parents who had experienced any of the aggressive acts was 74.2%.

In order of descending prevalence, the rate of experiencing each type of “acts of violence” was as follows: threatening with knife (15.1%), beating with an object (13.9%), visited physician for injury (10.8%), choking (6.0%), and injured with a knife (2.6%). On all items except for choking, parents with high IES-R scores had significantly more experiences of violence than parents with low IES-R scores. The percentage of parents who had ever experienced any “acts of violence” was 28.4%.

#### Risk factors related to IES-R

A multiple logistic regression was conducted with high- and low-IES-R groups as the dependent variable, and the types of violence experienced as the independent variables. High-IES-R was significantly greater for parents who had experienced an act of violence (OR = 2.03; 95% CI 1.09–3.80; using never experienced as a reference) and for the parents of patients who were hospitalized at the time of survey (OR = 2.47; 95% CI 1.01–6.06; using regularly visit psychiatrist as reference; Table 3).

**Table 3:** Factors related to parents’ risk of PTSD (n = 329).

		OR	95% CI	*P*
Type of family experienced violence				
Psychological violence (> 5 time)	N/A	1.00	reference	
	Yes	1.86	0.95–3.63	.069
Other aggressive acts (>1 time)	Never	1.00	reference	
	Ever	1.50	0.65–3.44	.343
Acts of violence (>1 time)	Never	1.00	reference	
	Ever	2.03	1.09–3.80	.027
Parents’ factors				
Primary caregiver	Yes	1.56	0.83–2.94	.171
	No	1.00	reference	
Patients’ factors				
Years since onset	(1-increment)	1.01	0.98–1.05	.512
Psychiatrist visit	Regularly	1.00	reference	
	Hospitalized	2.47	1.01–6.06	.048
	Not regularly	0.60	0.10–3.51	.575
Medication as instructed	Yes	1.00	reference	
	No	2.46	0.71–8.51	.154
Number of hospitalizations	0	1.00	reference	
	1–2	0.88	0.40–1.93	.743
	3 or more	0.89	0.37–2.15	.791
Rehabilitation	Yes	1.00	reference	
	No	1.74	0.95–3.18	.072

CI: Confidence interval.

## Discussion

### Risk of PTSD following familial violence

A multiple logistic regression revealed a significant relationship between high IES-R scores and the experience of familial violence. Univariate analysis found that parents in the high-IES-R group had experienced significantly more “acts of violence,” “other aggressive acts” and “psychological violence” than the low-IES-R group. However, the multivariate analysis found that the significant relationship was only present with “acts of violence,” types of physical violence in the current study, which were likely to result in severe injury and were more severe than “other aggressive acts” that were not likely to result in severe injury. This finding may mean that more severe violence has a stronger impact on the risk of PTSD. Therefore, it is imperative for severe family violence to be prevented in order to ameliorate the risk of developing PTSD in parents. The Japanese government has passed a law related to persons with mental illness who have committed serious criminal offences. However, there are reportedly few support programs for family members of such patients in Japan. Based on the results of the current study, family members of patients who commit severe violence need to receive mental health support and education, as it is not uncommon for family members of patients with schizophrenia to experience severe violence and to be at risk for PTSD.

### Risk of PTSD following hospitalization

A multiple logistic regression analysis revealed a significant relationship between high IES-R scores and patient hospitalization. This finding is consistent with the study by Kajitani and colleagues [8] who found that 58% of parents or siblings of patients who had undergone long-term hospitalization were at a higher risk of developing PTSD. According to a national survey conducted by family self-help groups, when the condition of a patient worsened, two-thirds of family members (64.8%) reported fearing that trouble would happen, one-third (30.9%) felt that they were in greater physical danger, and over one-half (58.7%) were concerned for their own mental and physical condition [17]. During crisis periods that occur prior to hospitalization, family members may experience traumatic events. Such traumatic events may be risk factors for PTSD. Moreover, the parents of inpatients at high risk for PTSD may have struggled with past experiences of violence committed by the patients and these parents likely did not receive any support or treatment for dealing with their stress and anxiety. Insufficient support or treatment for parents may make them hesitate to agree to the discharge of their own child from the hospital. Therefore, parents’ resisting the discharge of their child [10] results in challenges to achieving deinstitutionalization.

### Implications for practice

In the present study, the experience of serious violence and patient hospitalization was found to be related to a high risk of PTSD in parents. Both of these factors may be considered traumatic events that arise from crisis situations. In Japan, unlike the United States and Europe, there are few crisis intervention programs that provide a multidisciplinary team of specifically trained staff available 24 hours per day, who can promptly detect the exacerbation of serious mental illness and deliver swift, intense treatment in a community setting [18]. Furthermore, there are few opportunities for respite care for family members. Considering our findings, crisis intervention services need to be made more widely available in Japan. Moreover, the provision of support, education, or treatment for parents while their child is hospitalized is recommended.

### Research limitations and further research

There were some limitations to this study. First, the study sample only included parents from households belonging to family self-help groups. Therefore, the results do not offer any information on parents who do not participate in family groups. Such parents are less likely to have enough information on mental health treatment and services or the opportunity to learn about their child’s illness. Therefore, parents who do not participate in self-help groups may have greater burden and stress which may affect their own mental health, and these parents may also be at greater risk of violence. Second, most of the parents in this study cared for adult children who lived within the community. Consequently, this study offers only limited implications for parents of inpatients. The mental health of parents who care for inpatients requires further investigation. Parents at high risk for PTSD need to be supported, and such support has important implications not only for parents but also for the acceleration of deinstitutionalization.

## Supporting information

S1 Questionnaire (PDF)

S2 Questionnaire (PDF)
