# Supplementary material for: Post-traumatic stress disorder in parents of patients with schizophrenia following familial violence
Source: PLoS One. 2018 Jun 1;13(6):e0198164. doi: 10.1371/journal.pone.0198164 (PMC5983498; doi:10.1371/journal.pone.0198164)
Supplement: S1 Questionnaire — (PDF) [file pone.0198164.s001.pdf]

ID	－ －

あなたが支援されている精神障がいをお持ちの当事者（以下、当事者）の方について

あなたが支援する当事者本人の現在の状況について伺います。

1	当事者の年齢・性別	（　　　）歳　①男性・②女性　（どちらかに〇）
2	当事者の続柄	一つに〇（あなたからみて）<br>あなたの　①子　②配偶者　③兄弟姉妹<br>④その他（　　　　）
3	現在の主たる病名<br>（一つに〇）	①統合失調症、②うつ病、③躁うつ病、④発達障害、<br>⑤その他［医師からどのように説明を受けているかお書きください　］
4	発病時年齢・初診時年齢	発症推定　（　　）歳頃・初診時年齢　（　　　）歳頃
5	精神科での治療状況<br>（一つに〇）	①定期的に通院　②不定期に通院<br>③しばらく通院していない　④入院中<br>⑤まだ治療を受けたことはない（未受診）
6	精神科治療薬の服薬状況<br>（一つに〇）	①医師の指示通り服薬している<br>②医師の指示通りではないが服薬している<br>③服薬していない
7	これまでの入院回数	約（　　　）回
8	現在の社会参加状況<br>（一つに〇）	①ほとんど自宅で過ごす（家事、育児を含む）<br>②障がい者向けの施設に通所（就労支援は受けていない）<br>（デイケア、地域活動支援センター等）<br>③障がい者向けの就労支援を受けている<br>④一般就労（障がい者向けではない企業など）<br>⑤就学　⑥入院中<br>⑦その他［具体的にお書きください　］

あなたご自身について現在の状況を伺います。

あなたの年齢などの基本的な状況を教えてください。

1	年齢・性別	（　　　）歳　①男性・②女性　（どちらかに〇）
2	あなたの続柄<br>（一つに〇）	（当事者からみて）<br>当事者の　①父親　②母親　③夫　④妻<br>⑤兄　⑥弟　⑦姉　⑧妹　⑨子<br>⑩義理の兄弟姉妹　⑪その他（　　　　）
3	あなたと当事者の現在の同居<br>（住民票の世帯ではなく実際の状況）<br>（一つに〇）	①当事者と同居している<br>②当事者と同居していない
4	当事者の日常生活を<br>最も支援している人（一つに〇）	① あなた　②あなた以外
5	あなたの世帯収入（年収）<br>（生計を伴にする家族全員分、一つに〇）	①200 万円未満　②200 万円以上 400 万円未満<br>③400 万円以上 600 万円未満　④600 万円以上

あなたご自身は、過去 30 日の間にどれくらいの頻度で次のことがありましたか。当てはまる欄の数字に〇をつけてください。

		全くなし	少しだけ	ときどき	たいてい	いつも
1	神経過敏に感じましたか	0	1	2	3	4
2	絶望的だと感じましたか	0	1	2	3	4
3	そわそわ、落ち着かなく感じましたか	0	1	2	3	4
4	気分が沈み込んで、何が起こっても気が晴れないように感じましたか	0	1	2	3	4
5	何をするのも骨折りだと感じましたか	0	1	2	3	4
6	自分は価値のない人間だと感じましたか	0	1	2	3	4

次に、あなたやご家族が当事者から受けた暴力・暴言について伺います

<u>これまでの生涯</u>にあなた自身は、当事者から暴力・暴言をどのくらい受けましたか。

		これまでの生涯（過去１年間の回数含め）（一つに○）			
		全くない	１～４回	５～９９回	１００回以上
1	あなたに大声をあげたり、怒鳴った	0	1	2	3
2	病気になったのは、あなたのせいだと言った	0	1	2	3
3	あなたをけなした・見下した・侮辱した	0	1	2	3
4	あなたを「殺す」と言った	0	1	2	3
5	あなたを殴るそぶりや、蹴るそぶりをして脅した	0	1	2	3
6	あなたの体を押した・壁に押しつけた	0	1	2	3
7	あなたを手で叩いた・手で殴った・足で蹴った	0	1	2	3
8	あなたの首を絞めた	0	1	2	3
9	物を投げた・壊した	0	1	2	3
10	物をあなたに向かって投げた	0	1	2	3
11	物を使ってあなたを殴った	0	1	2	3
12	包丁などの鋭利な刃物をあなたに向けた	0	1	2	3
13	包丁などの鋭利な刃物であなたの体を傷つけた	0	1	2	3
14	**あなた自身が**暴力による外傷のために医療機関を受診した	0	1	2	3

下記の項目はいずれも、強いストレスを伴うような出来事に巻き込まれた方々に、後になって生じることのあるものです。あなたが経験した前問のような<u>当事者の精神疾患に関連する強いストレスを伴った出来事について</u>、本日を含む最近の１週間では、それぞれの項目の内容について、どの程度強く悩まされましたか。当てはまる欄に○をつけてください。（なお答えに迷われた場合は、不明とせず、もっとも近いと思うものを選んでください。）

	（最近の１週間の状態についてお答えください、このアンケートの回答中も含みます）	0.<br>全く<br>なし	1.<br>少し	2.<br>中<br>くらい	3.<br>かなり	4.<br>非常に
1	どんなきっかけでも、そのことを思い出すと、そのときの気もちがぶりかえしてくる					
2	睡眠の途中で目がさめてしまう					
3	別のことをしていても、そのことが頭から離れない					
4	イライラして、怒りっぽくなっている					
5	そのことについて考えたり思い出すときは、なんとか気を落ちつかせるようにしている					
6	考えるつもりはないのに、そのことを考えてしまうことがある					
7	そのことは、実際には起きなかったとか、現実のことではなかったような気がする					
8	そのことを思い出させるものには近よらない					
9	そのときの場面が、いきなり頭にうかんでくる					
10	神経が過敏になっていて、ちょっとしたことでどきっとしてしまう					
11	そのことは考えないようにしている					
12	そのことについては、まだいろいろな気持があるが、それには触れないようにしている					
13	そのことについての感情は、マヒしたようである					
14	気がつくと、まるでそのときにもどってしまったかのように、ふるまったり感じたりすることがある					
15	寝つきが悪い					
16	そのことについて、感情が強くこみあげてくることがある					
17	そのことを何とか忘れようとしている					
18	ものごとに集中できない					
19	そのことを思い出すと、身体が反応して、汗ばんだり、息苦しくなったり、むかむかしたり、どきどきすることがある					
20	そのことについての夢を見る					
21	警戒して用心深くなっている気がする					
22	そのことについては話さないようにしている					
