# Supplementary material for: Post-traumatic stress disorder in parents of patients with schizophrenia following familial violence
Source: PLoS One. 2018 Jun 1;13(6):e0198164. doi: 10.1371/journal.pone.0198164 (PMC5983498; doi:10.1371/journal.pone.0198164)
Supplement: S2 Questionnaire — (PDF) [file pone.0198164.s002.pdf]

ID	－ -

About the patient with mental disorder who you care for

Please answer about current situtation of the patient who you care for.

1	Age and gender	（ ） years old<br>Male or female（Select one）
2	Relationship to the patient	The patient is your<br>1. Child, 2.Spouse, 3.Sibling, 4, Other ( )
3	Main diagnosis	1. Schizophrenia, 2. Depression, 3. Bipolar disorder,<br>4. Developmental disorder, 5. Other ( )
4	Age of onset,<br>age of first visit for psychiatrist	Onset of the mental illness ( ) years old<br>First visit for psychiatrist ( ) years old
5	Psychiatrist visit	1. Visit regulary<br>2. Visit unregulary<br>3. Have not visited<br>4. Hospitalized<br>5. Have not visit ever
6	Medication as prescribed	1. Take medication as prescribed<br>2. Take medication as not prescribed<br>3. Have not taken medication
7	Number of hospitalization	( ) times
8	Current status of rehabilitation	1. Not using rehabilitation and spent almost time at home<br>2. Using rehabilitation for persons with disorders<br>3. Using rehabiliation for working<br>4. Working<br>5. Going school<br>6. Hospitalization<br>7. Other ( )

About respondent’s current situtation

Please anser your basic information.

1	Age and gender	（　　　）years old<br>Male or female（Select one）
2	Relationship to the patient	Your are the patient’s<br>1. Father 2. Mother 3. Husband 4. Wife<br>5. Older brother 6. Younger brother 7. Older sister<br>8.Younger sister 9. Child 10. Sibling-in-law<br>11. Other (　　)
3	Cohabitation with patient	1. living with the patient<br>2. Not living with the patient
4	Main caregiver	1. You 2. Other than you
5	Household income	1. less than ￥2,000,000<br>2. ￥2,000,000 to ￥4,000,000<br>3. ￥4,000,000 to ￥6,000,000<br>4. ￥6,000,000 or more

During the last 30 days, how often did…

		Non of the time				All ot the time
1	You feel nervous?	0	1	2	3	4
2	You feel hopeless?	0	1	2	3	4
3	You feel restless or fidgety?	0	1	2	3	4
4	You feel so depressed that nothing could cheer you up?	0	1	2	3	4
5	You feel that everythins was an effort?	0	1	2	3	4
6	You feel worthless?	0	1	2	3	4

About violence by patients

Among past one year and lifetime, what kind of violence and how many times have you experienced?

		lifetime			
		Never	1-4 times	5-99 times	100 times or more
1	Shouting	0	1	2	3
2	Blaming 'my illness is your fault'	0	1	2	3
3	Swearing and insulting	0	1	2	3
4	Saying 'I will kill you'	0	1	2	3
5	Gesturing of punching or kicking	0	1	2	3
6	Pushing	0	1	2	3
7	Punching and kicking	0	1	2	3
8	Choking	0	1	2	3
9	Destroyed property	0	1	2	3
10	Throwing an object	0	1	2	3
11	Beating with an object	0	1	2	3
12	Threatening with knife	0	1	2	3
13	Injured with knife	0	1	2	3
14	Visited physician for injury	0	1	2	3

INSTRUCTIONS: Below is a list of difficulties people sometimes have after stressful life events. Please read each item, and then indicate how distressing each difficulty has been for you DURING THE PAST SEVEN DAYS with respect to highly stressful events related patient with mental illness How much have you been distressed or bothered by these difficulties?

		0. Not at all	1. A little bit	2. Moderately	3. Quite a bit	4. Extremely
1	Any reminder brought back feelings about it.					
2	I had trouble staying asleep.					
3	Other things kept making me think about it.					
4	I felt irritable and angry.					
5	I avoided letting myself get upset when I thought about it or was reminded of it.					
6	I thought about it when I didn't mean to.					
7	I felt as if it hadn't happened or wasn't real.					
8	I stayed away from reminders of it.					
9	Pictures about it popped into my mind.					
10	I was jumpy and easily startled.					
11	I tried not to think about it.					
12	I was aware that I still had a lot of feelings about it, but I didn't deal with them.					
13	My feelings about it were kind of numb.					
14	I found myself acting or feeling like I was back at that time.					
15	I had trouble falling asleep.					
16	I had waves of strong feelings about it.					
17	I tried to remove it from my memory.					
18	I had trouble concentrating.					
19	Reminders of it caused me to have physical reactions, such as sweating, trouble breathing, nausea, or a pounding heart.					
20	I had dreams about it.					
21	I felt watchful and on-guard.					
22	2. I tried not to talk about it.					
